# Functional interplay between TTP/ZFP36 and HuR/ELAVL1 in cancer: a post-transcriptional regulatory network

**DOI:** 10.3389/fonc.2026.1913053

**Published:** 2026-07-20

**Authors:** Roberta Lotti, Tommaso Selmi, Alexis Grande, Tommaso Zanocco-Marani

**Affiliations:** 1 DermoLAB, Department of Surgical, Medical, Dental and Morphological Science (CHIMOMO), University of Modena and Reggio Emilia, Modena, Italy; 2 Consiglio Nazionale delle Ricerche, Istituto di Tecnologie Biomediche, Milano, Italy; 3 Department of Biomedical, Metabolic and Neural Sciences, University of Modena and Reggio Emilia, Modena, Italy; 4 Department of Life Sciences, University of Modena and Reggio Emilia, Modena, Italy

**Keywords:** AU-rich elements, cancer, ELAVL1, HuR, mRNA stability, N6-methyladenosine, RNA-binding proteins, tristetraprolin

## Abstract

Post-transcriptional regulation of gene expression has emerged as a fundamental determinant of cancer initiation, progression, and therapeutic response. Among the RNA-binding proteins (RBPs) involved in mRNA turnover and translational regulation, tristetraprolin (TTP), encoded by the *ZFP36* gene, and “Human antigen R” (HuR), encoded by *ELAVL1*, represent two functionally antagonistic regulators of AU-rich element (ARE)-containing transcripts. TTP promotes the degradation of target mRNAs through recruitment of deadenylation and decay complexes, whereas HuR generally stabilizes and enhances the translation of overlapping mRNA subsets. Because many oncogenic, inflammatory, angiogenic, and metastasis-associated transcripts contain AREs within their 3′ untranslated regions, the balance between TTP-mediated decay and HuR-mediated stabilization critically influences tumor biology. Accumulating evidence demonstrates that loss of TTP expression or activity and cytoplasmic accumulation of HuR are recurrent features across multiple cancer types, including breast, colorectal, pancreatic, gastric, liver, ovarian, and lung cancers. Importantly, several studies indicate that the reciprocal interplay between these proteins establishes a post-transcriptional rheostat controlling cancer-associated RNA regulons. This review summarizes current knowledge regarding the molecular biology of TTP and HuR, emphasizing their opposing functions in mRNA metabolism and cancer progression. We discuss mechanisms regulating their expression, localization, phosphorylation, and RNA-binding activity; analyze cancer-specific evidence; and examine models in which both proteins are co-expressed or functionally interconnected. Finally, we evaluate therapeutic strategies aimed at restoring TTP function or inhibiting HuR activity and discuss future perspectives for targeting post-transcriptional regulatory networks in oncology.

## Introduction

The regulation of gene expression extends far beyond transcriptional control and includes multiple post-transcriptional mechanisms governing mRNA splicing, transport, localization, stability, and translation. RNA-binding proteins (RBPs) constitute a major class of regulatory molecules orchestrating these processes by interacting with specific RNA motifs and recruiting enzymatic complexes that determine transcript fate. Dysregulation of RBPs is increasingly recognized as a hallmark of cancer, contributing to aberrant proliferation, resistance to apoptosis, immune evasion, angiogenesis, epithelial-to-mesenchymal transition (EMT), and metastatic dissemination.

A central post-transcriptional mechanism in mammalian cells involves the recognition of AU-rich elements (AREs), which are commonly located in the 3′ untranslated regions (3′UTRs) of mRNAs encoding cytokines, proto-oncogenes, growth factors, cyclins, and transcription factors. ARE-containing transcripts are inherently unstable and tightly regulated under physiological conditions. Two RBPs with opposite effects on ARE-containing transcripts are Tristetraprolin (TTP) and “Human antigen R” (HuR).

Tristetraprolin (TTP) belongs to the ZFP36 family of CCCH tandem zinc finger proteins and promotes rapid mRNA degradation by binding AREs and recruiting deadenylation machinery, decapping complexes, and exonucleases (1–3). In most contexts, TTP acts as a tumor suppressor by destabilizing transcripts encoding inflammatory mediators and oncogenic proteins (4, 5).

Conversely, HuR, a ubiquitously expressed member of the embryonic lethal abnormal vision-like (ELAV-like) protein family, typically stabilizes target mRNAs and enhances their translation (6–8). Cytoplasmic translocation of HuR is frequently associated with malignant transformation and poor prognosis (9–11). HuR supports tumor cell survival through stabilization of mRNAs encoding cyclins, anti-apoptotic proteins, angiogenic mediators, inflammatory cytokines, and EMT regulators.

Because TTP and HuR frequently target overlapping sets of ARE-containing transcripts, their relative abundance and activity determine the stability and translational output of key cancer-associated mRNAs (12). In many tumors, reduced TTP expression or activity occurs simultaneously with elevated HuR expression or cytoplasmic localization, thereby shifting post-transcriptional regulation toward oncogenic transcript stabilization (13).

This review analyzes the opposing biological roles of TTP and HuR in cancer, examines the evidence supporting a coordinated post-transcriptional network involving these RBPs and evaluates therapeutic strategies aimed at manipulating this axis.

## Structural and functional features of TTP and HuR

TTP is encoded by the *ZFP36* gene located on chromosome 19q13.1. The protein contains two tandem CCCH zinc finger domains that specifically recognize AU-rich sequences within the 3′ untranslated regions (UTRs) of target mRNAs. Binding of TTP to these regions promotes transcript degradation through recruitment of the CCR4-NOT deadenylase complex, decapping enzymes, and exonucleases (14). Through this mechanism, TTP drives rapid turnover of transcripts encoding inflammatory cytokines, growth factors, proto-oncogenes, and other tightly regulated proteins.

The biological activity of TTP is strongly modulated by post-translational modifications (1, 3). Inflammatory and stress-associated signaling pathways, particularly p38 MAPK/MK2 signaling, induce phosphorylation of the protein and impair its RNA decay activity (15–17). Thus, although TTP expression may initially increase during inflammatory responses, persistent activation of oncogenic signaling pathways frequently renders the protein functionally inactive. This phenomenon appears particularly relevant in cancer, where sustained inflammatory signaling contributes to stabilization of oncogenic transcripts.

HuR contains three RNA recognition motifs that enable high-affinity binding to AU- rich elements. Under basal conditions, HuR predominantly localizes within the nucleus, but cellular stress, DNA damage, hypoxia, and oncogenic activation promote its translocation into the cytoplasm. Cytoplasmic HuR stabilizes target mRNAs and frequently enhances their translation. HuR regulates extensive RNA networks associated with proliferation, survival, angiogenesis, epithelial-to-mesenchymal transition (EMT), stemness, and immune modulation (7, 12, 18). Important targets include cyclin D1, cyclin E1, VEGF, COX-2, HIF-1α, BCL2, Snail, and β-catenin (19–25). Cytoplasmic accumulation of HuR has been associated with poor prognosis in several malignancies and is increasingly recognized as a marker of aggressive disease.

Hur exerts effects that are largely opposite to those mediated by TTP considering their convergence over functionally related RNA networks containing either HuR or TTP binding sites. TTP and HuR also regulate overlapping transcriptomes, specifically by competing for the same AU-rich sequences on a set of at least 59 target mRNAs (26). Common targets include several cytokines like TNF-alpha, GM-CSF and IL-10; cancer-associated genes like COX-2, VEGF, beta-catenin, SNAIL and TWIST, among many others. Consequently, the biological fate of many cancer-associated transcripts depends on the dynamic balance between destabilizing and stabilizing post-transcriptional forces.

The antagonistic relationship between TTP and HuR represents an interesting example of coordinated post-transcriptional regulation in mammalian cells. AU-rich elements are present in a substantial proportion of transcripts encoding cytokines, transcription factors, growth factors, and proto-oncogenes, allowing rapid adaptation of gene expression programs to environmental stimuli. Under physiological conditions, this system enables transient expression of inflammatory and stress-response genes followed by prompt transcript degradation once homeostasis is restored. The relative abundance, localization, and activation state of HuR and TTP therefore determine whether a transcript undergoes rapid decay or persistent stabilization.

Several oncogenic transcripts appear to be regulated through this competitive mechanism, including TNF-α, IL-6, IL-8, COX-2, VEGF, cyclin D1, c-Myc, BCL2, and HIF-1α (12, 27–30). In cancer cells, chronic inflammatory signaling often shifts the balance toward HuR-dependent stabilization (31, 32). Activation of p38/MK2 signaling suppresses TTP-mediated mRNA decay while simultaneously promoting cytoplasmic accumulation of HuR (33, 34). Similarly, PI3K/AKT, ERK, and NF-κB pathways reinforce stabilization of inflammatory and oncogenic transcripts through HuR (35, 36).

An additional level of complexity derives from reciprocal regulation between the two proteins themselves. TTP has been reported to destabilize ELAVL1/HuR mRNA, thereby limiting HuR abundance (37). Conversely, HuR stabilizes transcripts encoding inflammatory mediators and signaling molecules that indirectly suppress TTP expression or activity (38). There is substantial evidence that TTP can directly regulate HuR expression, while reciprocal HuR-mediated regulation of TTP is less firmly established and may be more indirect or context-specific. The deregulation of these interactions contributes to the formation of a self-reinforcing post-transcriptional circuit that promotes chronic inflammation and tumor progression.

Rather than functioning independently, TTP and HuR therefore appear to constitute opposing components of an integrated post-transcriptional regulatory axis.

## Role of TTP in cancer

Accumulating evidence supports a predominantly tumor-suppressive role for TTP in human malignancies (5, 39–42). Reduced expression or impaired activity of the protein has been documented in breast, colorectal, pancreatic, liver, ovarian, prostate, and lung cancers. Because TTP controls numerous inflammatory and oncogenic transcripts simultaneously, its loss produces widespread alterations in cellular behavior.

One of the earliest recognized functions of TTP was the regulation of inflammatory cytokines such as TNF-α (43). Mice lacking TTP develop severe systemic inflammation due to uncontrolled cytokine production, highlighting the importance of this protein in maintaining immune homeostasis (1, 44, 45). Similar mechanisms are highly relevant in cancer, where persistent inflammation contributes to tumor initiation, progression, and metastatic dissemination.

By destabilizing transcripts encoding IL-6, IL-8, COX-2, VEGF, and GM-CSF, TTP limits the establishment of protumorigenic inflammatory microenvironments. Loss of TTP therefore enhances recruitment of inflammatory cells, angiogenesis, stromal remodeling, and cytokine-driven survival signaling.

TTP also exerts important antiproliferative effects through destabilization of transcripts encoding cyclin D1, c-Myc, E2F1, and related cell cycle regulators (46, 47). Experimental restoration of TTP expression frequently suppresses proliferation, clonogenicity, migration, and invasion while increasing apoptotic sensitivity. In breast and colorectal cancer models, re-expression of TTP markedly reduces aggressive phenotypes.

The protein additionally participates in the control of epithelial-to-mesenchymal transition. Destabilization of SNAIL, TWIST, ZEB1, MMP2, and MMP9 transcripts contributes to suppression of migration and metastatic dissemination (48, 49). Consequently, tumors characterized by low TTP expression often exhibit increased invasiveness and poor differentiation.

In addition, deregulated TTP can influence inflammasomes activity; inflammasomes are multi-protein complexes that detect tissue damage and initiate innate immune responses; they can suppress cancer by triggering anti-tumor immunity and specialized cell death. It has been demonstrated that TTP can target the NLRP3 inflammasome, thereby influencing its activity (50, 51).

Importantly, many tumors do not simply eliminate TTP expression but rather impair its function through persistent signaling-dependent phosphorylation. Continuous activation of the p38MAPK/MK2 pathway stabilizes phosphorylated TTP while reducing its RNA decay activity (15, 17). Thus, detectable TTP protein expression does not necessarily indicate preserved tumor-suppressive function. Moreover, deregulated pathways underlying cancer can lead to TTP downregulation in absence of a gene silencing mutation, as is the case of WNT/Beta-Catenin in squamous cell carcinoma (49).

Additional mechanisms contributing to TTP suppression include promoter methylation (51), oncogenic Ras signaling leading to TTP phosphorylation/inactivation (52), and microRNA-mediated repression (53). Together, these alterations shift post-transcriptional regulation toward persistent stabilization of inflammatory and oncogenic transcripts.

## Role of HuR in cancer

In contrast to TTP, HuR generally functions as a pro-oncogenic RNA-binding protein (6, 8, 54). Increased cytoplasmic HuR expression has been associated with poor prognosis and aggressive behavior in multiple malignancies, including colorectal, pancreatic, breast, ovarian, gastric, liver, and lung cancers.

The oncogenic activity of HuR derives from its ability to stabilize and enhance translation of transcripts involved in proliferation, angiogenesis, survival, DNA repair, stress adaptation, and metastasis. Cytoplasmic localization is particularly important because HuR exerts most of its stabilizing activity outside the nucleus.

HuR promotes proliferation through stabilization of cyclin D1, cyclin E1, c-Fos, c-Myc, and related transcripts. In pancreatic ductal adenocarcinoma and colorectal cancer, high cytoplasmic HuR expression correlates strongly with aggressive disease and unfavorable clinical outcome (55, 56).

Another major function of HuR involves adaptation to stress conditions such as hypoxia and chemotherapy exposure. Stabilization of VEGF and HIF-1α transcripts supports angiogenesis and cellular adaptation to oxygen deprivation (57), whereas stabilization of anti-apoptotic transcripts such as BCL2 and MCL1 promotes survival under therapeutic stress (58).

HuR has also emerged as an important mediator of therapy resistance. In pancreatic cancer, regulation of deoxycytidine kinase by HuR influences gemcitabine responsiveness (59, 60). In additional tumor models, HuR contributes to resistance against cisplatin, tamoxifen, and radiotherapy through stabilization of transcripts involved in DNA repair and stress adaptation.

The protein further contributes to EMT and metastatic progression through stabilization of Snail, MMP9, β-catenin, and TGF-β-associated transcripts (22, 24, 61–63). Elevated cytoplasmic HuR therefore frequently correlates with metastatic dissemination.

Because many HuR targets overlap with transcripts regulated by TTP, the oncogenic effects of HuR are particularly pronounced in tumors where TTP activity is simultaneously reduced.

## Functional interplay between TTP and HuR in cancer

The extensive overlap between transcripts targeted by TTP and HuR is a compelling aspect of post-transcriptional regulation in cancer. Shared targets include TNF-α, COX-2, VEGF, IL-6, IL-8, cyclin D1, c-Myc, BCL2, MMP9, and HIF-1α, SNAIL, TWIST, among many others, all of which play central roles in inflammation, proliferation, angiogenesis, apoptosis resistance, and metastasis.

Under physiological conditions, the interplay between TTP and HuR allows transient stabilization of stress-response transcripts followed by rapid degradation once the stimulus resolves. In cancer, however, this equilibrium is frequently disrupted. Reduced TTP expression or activity often occurs simultaneously with increased HuR cytoplasmic localization, creating a post-transcriptional environment that strongly favors stabilization of oncogenic transcripts.

Breast cancer provides a representative example of this imbalance. Several studies have demonstrated reduced TTP expression together with elevated HuR activity, resulting in enhanced inflammatory and proliferative signaling (9, 19, 25, 29, 63–65). Restoration of TTP expression antagonizes HuR-driven oncogenic programs and suppresses migration and invasion.

A similar pattern has been observed in colorectal cancer, where inflammatory signaling promotes HuR cytoplasmic accumulation and suppresses TTP activity (30, 56). Shared regulation of COX-2 appears particularly important in this context because HuR-mediated stabilization of COX-2 mRNA supports persistent inflammatory signaling, whereas TTP normally acts to terminate the response.

Pancreatic ductal adenocarcinoma is a further example of HuR-dependent malignancy. Cytoplasmic HuR strongly contributes to chemoresistance and survival signaling, while reduced TTP activity further enhances stabilization of inflammatory and anti-apoptotic transcripts (41, 60, 66).

In hepatocellular carcinoma (65, 67) and gastric cancer (35, 68), chronic inflammatory conditions similarly appear to shift the balance toward HuR-dependent stabilization and away from TTP-mediated decay. Collectively, these observations support the concept that TTP and HuR function as a post-transcriptional rheostat controlling the fate of cancer-associated RNA regulons.

Multiple signaling pathways converge on the TTP–HuR axis and profoundly influence the stability of cancer-associated transcripts (10, 16, 44). Among these pathways, p38 MAPK/MK2 signaling occupies a particularly central role. Activation of MK2 promotes phosphorylation of TTP, thereby impairing its mRNA decay activity, while simultaneously enhancing inflammatory transcript persistence and HuR cytoplasmic accumulation (16, 17, 33, 69, 70). This dual effect is especially relevant in tumors characterized by chronic inflammation. Persistent inflammatory signaling shifts post-transcriptional regulation away from rapid transcript turnover and toward sustained expression of cytokines, growth factors, angiogenic mediators, and survival proteins. NF-κB signaling further amplifies these effects because HuR stabilizes cytokine transcripts capable of sustaining NF-κB activation, whereas ZFP36 normally destabilizes many NF-κB target mRNAs (35, 35, 71, 72).

Hypoxia constitutes another major regulatory condition. Hypoxic stress promotes HuR cytoplasmic translocation and stabilization of HIF-1α and VEGF transcripts, thereby facilitating angiogenesis and adaptation to oxygen deprivation (20, 57, 73, 74). TTP can counteract these responses through destabilization of angiogenic transcripts (13, 75–77), although this activity is often impaired in aggressive tumors.

Together, these signaling pathways integrate extracellular stress, inflammation, oncogenic activation, invasion ability into coordinated post-transcriptional responses that strongly influence cancer progression.

## Interaction of the TTP–HuR axis with epitranscriptomic regulation and microRNAs

Accumulating evidence indicates that the antagonistic regulation exerted by TTP and HuR might be further modulated by additional layers of post-transcriptional control, such as N^6^-methyladenosine (m^6^A) RNA modification and microRNAs (miRNAs). Although these regulatory mechanisms have largely been investigated independently, recent studies suggest that they converge to determine the stability, translation and degradation of numerous cancer-associated transcripts.

Among epitranscriptomic modifications, N^6^-methyladenosine (m^6^A) has emerged as one of the most important determinants of mammalian mRNA metabolism. The deposition of m^6^A by the METTL3/METTL14 methyltransferase complex and its removal by the demethylases FTO and ALKBH5 regulate mRNA stability, translation and localization through the recruitment of specific reader proteins, including members of the YTH-domain and IGF2BP families (78, 79). Importantly, m^6^A also modulates the interaction between transcripts and conventional RNA-binding proteins. HuR represents a good example of this interplay. Rather than directly recognizing methylated adenosines, HuR preferentially binds RNA regions whose accessibility is altered by m^6^A-induced changes in RNA secondary structure, a mechanism known as the m^6^A switch (80, 81). Moreover, HuR binding may antagonize m^6^A-dependent transcript degradation by limiting the recruitment of decay-promoting proteins, thereby contributing to the stabilization of specific mRNAs (80). Conversely, m^6^A-mediated recruitment of IGF2BP proteins stabilizes numerous oncogenic transcripts and frequently complements the transcript-stabilizing activity exerted by HuR (82). In addition, recent evidence indicates that METTL3-dependent m^6^A methylation can functionally cooperate with HuR in cancer cells by promoting the stabilization and expression of oncogenic transcripts, further supporting the concept that m^6^A writers and conventional RNA-binding proteins act in concert to regulate tumor-associated gene expression (83). Since dysregulation of m^6^A writers, erasers and readers is common in many human cancers, alterations of the epitranscriptome are therefore likely to influence HuR-dependent post-transcriptional regulation and, consequently, tumor progression (78–85).

**Figure 1:**
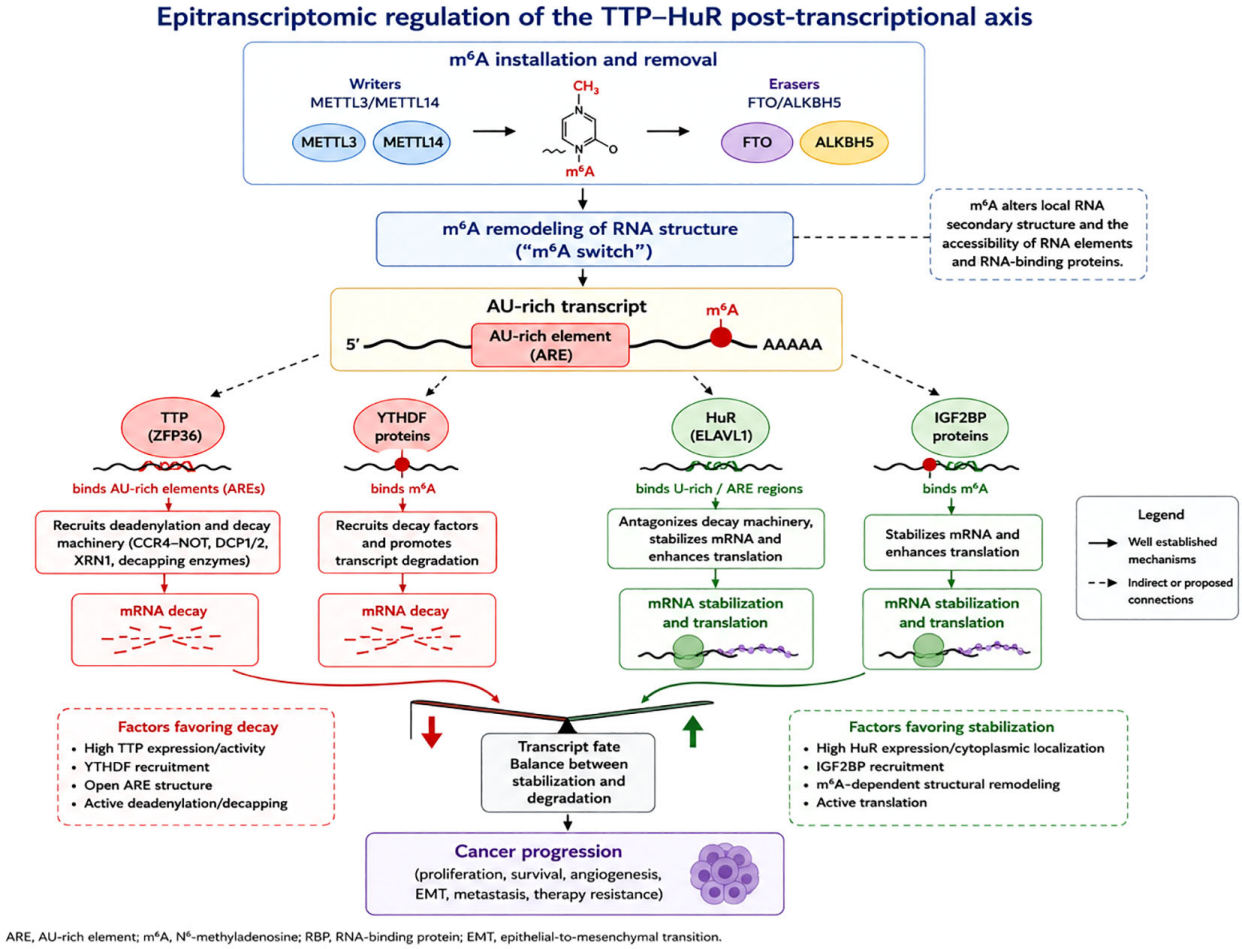
Epitranscriptomic regulation of the TTP–HuR post-transcriptional axis. m^6^A marks are deposited by the METTL3/METTL14 complex and removed by the demethylases FTO and ALKBH5. m^6^A-dependent remodeling of RNA secondary structure (the *m^6^A switch*) alters the accessibility of target transcripts to RNA-binding proteins (RBPs). TTP binds AU-rich elements (AREs) and promotes mRNA decay, whereas HuR stabilizes ARE-containing transcripts and enhances translation. m^6^A reader proteins of the IGF2BP and YTHDF families further regulate transcript stability by promoting mRNA stabilization or degradation, respectively. We propose that m^6^A-mediated structural remodeling of target mRNAs influences the accessibility of RBPs, thereby modulating the balance between TTP-mediated mRNA decay and HuR-mediated transcript stabilization.

Recent evidence further suggests that the relationship between m^6^A and the TTP–HuR axis extends beyond regulation of HuR alone. A recent transcriptome-wide study demonstrated that AU-rich elements (AREs) and neighboring m^6^A sites frequently coexist within rapidly decaying immune-related transcripts and cooperate in determining mRNA stability, indicating that local 3′UTR architecture represents a critical determinant of post-transcriptional regulation (86). Rather than acting as an isolated epitranscriptomic mark, m^6^A therefore appears to create a regulatory platform in which AU-rich elements, RNA secondary structure and multiple RNA-binding proteins collectively determine transcript fate. Within this framework, the biological output of the TTP–HuR axis depends not only on the relative abundance or activity of TTP and HuR, but also on the epitranscriptomic organization of individual transcripts, which influences the accessibility of AU-rich regions and the recruitment of stabilizing or destabilizing RNA-binding proteins. Consequently, m^6^A may indirectly shift the equilibrium between HuR-mediated transcript stabilization and TTP-mediated transcript decay without directly regulating either protein. This concept is consistent with the observation that m^6^A reader proteins such as IGF2BPs and decay-promoting readers such as YTHDF proteins can respectively reinforce transcript stabilization or degradation, thereby influencing the same post-transcriptional regulatory pathways controlled by HuR and TTP (78–82, 84). Although direct evidence linking TTP to m^6^A-dependent regulation remains limited and TTP has not been identified as a canonical m^6^A reader, current findings suggest that RNA methylation acts as an upstream determinant of the TTP–HuR regulatory axis by shaping the competition among RNA-binding proteins for shared target transcripts. Future studies combining transcriptome-wide mapping of m^6^A, RNA-binding protein occupancy and RNA stability will be required to determine whether m^6^A-dependent remodeling of RNA structure directly influences TTP recruitment or primarily regulates the dynamic balance between stabilizing and destabilizing ribonucleoprotein complexes. A schematic model summarizing the current understanding of how epitranscriptomic regulation may influence the functional balance between TTP- and HuR-mediated post-transcriptional control is presented in **Figure 1**.

A second level of regulation derives from the extensive interplay between TTP, HuR and miRNAs. Since AU-rich elements and miRNA recognition sites frequently coexist within the same 3′ untranslated regions, RNA-binding proteins and miRNAs may cooperate or compete to regulate transcript fate. HuR has been shown to either antagonize or facilitate miRNA-mediated repression depending on the target transcript and the cellular context. In several models, HuR binding adjacent to miRNA recognition sites prevents recruitment of Argonaute-containing RNA-induced silencing complexes (RISC), thereby protecting target mRNAs from miRNA-dependent degradation. Conversely, under different conditions, HuR may promote the association of specific miRNAs with their targets, highlighting its role as a context-dependent modulator rather than a simple antagonist of miRNA function. TTP also interacts functionally with the miRNA machinery. By recruiting deadenylation and decapping complexes, TTP cooperates with components of the RISC complex to accelerate degradation of shared target transcripts, particularly inflammatory cytokines and immediate-early response genes. Moreover, reciprocal regulatory loops exist between miRNAs and TTP expression, since several oncogenic miRNAs directly repress ZFP36, whereas TTP indirectly modulates miRNA expression by controlling inflammatory signaling pathways. Collectively, these observations indicate that TTP and HuR operate within a broader post-transcriptional regulatory network in which RNA-binding proteins and miRNAs act cooperatively to fine-tune gene expression rather than functioning as independent regulators. Such integrated regulation is likely to contribute to the context dependency of the TTP–HuR axis observed across different tumor types and may partly explain why modulation of either protein frequently produces distinct biological outcomes despite overlapping target repertoires (87–89).

## The exception to the rule: contexts in which TTP activity or HuR inactivation may sustain tumor progression

Although TTP is generally regarded as a tumor suppressor and HuR is typically considered pro-oncogenic, increasing evidence indicates that these functional assignments are not universally applicable. The biological consequences of TTP and HuR activity appear to depend strongly on cellular context, tumor type, metabolic state, inflammatory signaling, and the identity of the regulated transcriptome. Consequently, exceptions to the canonical TTP–HuR paradigm have begun to emerge, highlighting the complexity of post-transcriptional regulation in cancer biology.

One important concept is that RNA-binding proteins do not regulate exclusively oncogenic transcripts but instead control large and heterogeneous RNA regulons. Depending on the cellular environment, stabilization or destabilization of a given transcript subset may unexpectedly favor tumor adaptation, survival, or immune evasion. Recent studies therefore suggest that the biological outcome of TTP or HuR activity cannot always be predicted solely on the basis of their classical functions as mRNA destabilizer and stabilizer, respectively.

An emerging example concerns the context-dependent role of HuR in regulating tumor suppressive pathways. Although HuR most commonly stabilizes pro-oncogenic transcripts, several reports indicate that it may also stabilize mRNAs encoding proteins involved in growth inhibition or apoptosis. It was demonstrated that HuR can interfere with oncogenic microRNA activity and may stabilize tumor suppressor transcripts such as PDCD4 under specific stress conditions (89). In these settings, inhibition of HuR could theoretically favor malignant progression rather than suppress it.

Similarly, evidence is emerging that TTP activity may not always oppose tumor development. In bladder cancer it was demonstrated that enhanced TTP activity is linked to worse patient outcome (90). Likewise, in a non-small cell lung cancer (NSCLC) model, TTP was identified as a key determinant of resistance to KRAS inhibitors (91). On the other hand, in the same cancer context of NSCLC it was also suggested that enhancing the suppressive role of TTP might contribute to counteract chemoresistance (92). These observations further support the notion that the biological consequences of TTP activation depend on the cellular transcriptomic landscape rather than on a universally tumor-suppressive program.

Mechanistically, one explanation for these paradoxical observations is that TTP targets not only inflammatory and oncogenic mRNAs but also transcripts involved in senescence, apoptosis, interferon signaling, and immune activation. Excessive degradation of such transcripts could theoretically provide selective advantages to tumor cells, particularly under therapeutic or metabolic stress. Moreover, because TTP activity is strongly influenced by phosphorylation state and interacting cofactors, different forms of the protein may exert distinct biological effects in different cellular compartments.

Another important consideration is tumor heterogeneity. Cancer cells frequently undergo profound metabolic and epigenetic reprogramming, which alters the repertoire of accessible ARE-containing transcripts. Under these conditions, HuR inhibition may destabilize transcripts required for adaptation to oxidative stress or DNA damage in ways that paradoxically increase genomic instability and tumor evolution. Conversely, TTP activation could selectively eliminate transcripts involved in immune surveillance or differentiation, thereby favoring aggressive phenotypes in specific tumor contexts.

Collectively, these observations indicate that the TTP–HuR axis should not be interpreted as a strictly binary tumor suppressor–oncogene system. Instead, the biological consequences of TTP activation or HuR inhibition appear to depend on tumor lineage, stress conditions, inflammatory state, and transcriptomic context. These exceptions are particularly important from a therapeutic perspective because they suggest that pharmacological manipulation of RNA-binding proteins may require biomarker-guided patient stratification and careful characterization of the regulated RNA networks in individual tumor types.

Future studies combining CLIP-seq, single-cell transcriptomics, proteomics, and functional perturbation approaches will likely be required to clarify the circumstances under which TTP or HuR adopt noncanonical functions in cancer progression. Such investigations may ultimately reveal that the oncogenic or tumor-suppressive properties of these proteins are not intrinsic, but rather emerge from the dynamic architecture of the post-transcriptional networks in which they operate.

## TTP, HuR, and cancer immunotherapy

The increasing success of cancer immunotherapy has highlighted the importance of understanding the molecular mechanisms that regulate immune activation, T-cell persistence, and immune escape within the tumor microenvironment. Among the post-transcriptional regulators involved in these processes, TTP and HuR have emerged as important modulators of inflammatory signaling, T-cell function, and adaptive responses to immune pressure. Although these RNA-binding proteins were initially studied mainly in the context of inflammation and tumor cell biology, they might also play critical roles in determining the efficacy of modern immunotherapeutic approaches, including immune checkpoint blockade and CAR-T cell therapy.

Moreover, should the general idea that TTP might act as a tumor suppressor and HuR as an oncogenic activator in cancer cells be true, it is not definitely clear whether TTP and HuR activation/inactivation in immune cells might be an advantage or a disadvantage against cancer cells. As far as TTP is concerned, there is evidence that its activation in cancer cells leads to direct PDL1 downregulation, thereby improving sensitivity to immunotherapy (52, 93–95). On the other hand, it was investigated whether inactivation of TTP in CAR-T cells, leading to improved inflammatory function and cytokine production, might enhance antitumor activity. Results showed that this did not enhance overall performance both *in vitro* and *in vivo* (96). As far as HuR is concerned, studies suggest that HuR is responsible for PD-L1 stabilization, thereby reducing cells’ sensitivity to immunotherapy (94, 95). Collectively, these observations indicate that the role of TTP and HuR in cancer immunotherapy seems to be in accord with the traditional paradigm in which TTP acts as a tumor suppressor and HuR as an oncogenic factor. Nevertheless, their functions are dependent on the specific immune context, tumor type, and therapeutic modality involved, therefore research is still needed to clarify if TTP activity may always enhance antitumor immunity by improving T-cell persistence and function, whereas excessive HuR activation may always sustain immune resistance through stabilization of stress-adaptation and immunosuppressive transcripts.

## TTP and HuR as prognostic biomarkers in cancer

Beyond their mechanistic roles in tumor progression, TTP and HuR have also been extensively investigated as potential prognostic biomarkers in human malignancies. In general, reduced TTP expression and increased cytoplasmic HuR accumulation are associated with aggressive clinicopathological features and unfavorable clinical outcomes in several tumor types, including breast, colorectal, pancreatic, gastric, hepatocellular and ovarian carcinomas. Early immunohistochemical studies demonstrated that cytoplasmic, rather than nuclear, localization of HuR correlates with poor overall survival and increased metastatic potential, supporting the concept that HuR subcellular localization represents a functional indicator of its mRNA-stabilizing activity rather than simply a measure of protein abundance (9, 55). Similarly, reduced TTP expression has been associated with higher tumor grade, increased invasiveness and shorter patient survival in colorectal, pancreatic and head and neck cancers, consistent with its established tumor-suppressive functions (48, 64, 66). Nevertheless, the prognostic significance of both proteins is not universal. Several studies have reported weak or absent associations with clinical outcome, while others have identified prognostic value only in specific molecular subgroups or treatment settings. For example, in pancreatic ductal adenocarcinoma, high cytoplasmic HuR has paradoxically been associated with improved response to gemcitabine because HuR stabilizes the mRNA encoding deoxycytidine kinase, the enzyme responsible for intracellular activation of the drug (55, 59). Thus, although HuR generally promotes tumor progression, its expression may also predict sensitivity to specific therapeutic agents.

The prognostic interpretation of TTP is similarly influenced by biological context. Although decreased TTP expression is generally associated with poor prognosis, recent studies suggest that its clinical significance depends on the repertoire of regulated transcripts, the phosphorylation state of the protein and the signaling pathways active within individual tumors. Indeed, TTP expression does not necessarily reflect functional activity, since persistent activation of the p38–MK2 pathway can maintain TTP in an inactive phosphorylated state despite preserved protein levels. Moreover, recent mechanistic studies indicate that under specific transcriptional contexts TTP may regulate subsets of transcripts that support tumor progression, suggesting that its biological impact depends on the composition of the underlying RNA regulatory network rather than on expression levels alone (90, 91).

Collectively, these observations indicate that neither TTP nor HuR should currently be regarded as universal prognostic biomarkers. Rather, their clinical value appears to be highly context-dependent and influenced by tumor lineage, molecular subtype, treatment modality, subcellular localization, post-translational regulation and the composition of the RNA regulatory networks operating in individual cancers. Future studies integrating transcriptomic, proteomic and spatial analyses with clinical outcome data will be essential to determine whether these RNA-binding proteins can be incorporated into biomarker panels for patient stratification and therapeutic decision-making.

## TTP and HuR as therapeutic targets

The opposing biological functions of TTP and HuR make this regulatory axis an attractive therapeutic target in oncology. Because these proteins simultaneously regulate large networks of cancer-associated transcripts, pharmacological modulation of their activity may affect multiple hallmarks of cancer at once.

Restoration of TTP activity represents an attractive therapeutic strategy because TTP functions as a tumor suppressor in many malignancies. However, unlike HuR, no pharmacological agents capable of directly activating TTP have yet entered clinical development. Current approaches therefore focus on indirect restoration of TTP activity by targeting signaling pathways responsible for its functional inactivation or by increasing its expression (49, 97). Experimental strategies aimed at restoring TTP expression or activity have been shown to suppress tumor cell proliferation, inflammatory signaling, angiogenesis, invasion and metastatic dissemination in multiple preclinical cancer models (48, 64, 98–100). However, these approaches remain experimental and have not yet translated into clinically approved therapies. Inhibition of p38MAPK/MK2 signaling may be particularly beneficial because it can restore endogenous TTP activity while simultaneously reducing inflammatory signaling (3, 34, 98).

Epigenetic strategies may also prove useful in tumors where TTP expression is suppressed through promoter methylation or microRNA-mediated mechanisms. In addition, development of approaches capable of stabilizing the active dephosphorylated form of TTP may further enhance tumor-suppressive activity.

In parallel, HuR has emerged as one of the most intensively investigated RBPs in cancer therapeutics. Several small molecules capable of interfering with HuR RNA binding or cytoplasmic localization have demonstrated promising preclinical activity. Compounds such as MS-444, KH-3, and related inhibitors reduce proliferation, impair survival signaling, and enhance chemosensitivity in multiple tumor models. In particular, MS-444 attenuates invasion in a model of glioma (101), and promotes growth inhibition and apoptosis in colorectal cancer (56). On the other hand, HuR inhibitor KH-3 is capable to suppressing breast cancer invasion and metastasis (102), sensitizing breast cancer to chemotherapy (25) and inducing apoptosis in breast and prostate cancer (103).

The role of HuR in therapy resistance further increases its clinical relevance (18, 104–107). In pancreatic cancer and other aggressive malignancies, inhibition of HuR enhances responsiveness to cytotoxic agents and interferes with adaptive stress responses (59, 108–110). Because HuR controls extensive RNA networks involved in DNA repair and apoptosis resistance, therapeutic inhibition may sensitize tumors to conventional therapies.

An especially attractive possibility involves simultaneous targeting of both sides of the post-transcriptional rheostat. Combined restoration of TTP activity together with inhibition of HuR could shift ARE-mediated regulation toward degradation of oncogenic transcripts. Such approaches may prove particularly effective in tumors characterized by chronic inflammation and strong dependence on post-transcriptional stabilization programs.

The principal therapeutic strategies aimed at targeting the TTP–HuR axis in cancer are summarized in **Table 1**. Despite encouraging preclinical evidence, important challenges remain before these approaches can be translated into clinical practice. RNA-binding proteins have historically been considered difficult pharmacological targets because of their structural flexibility and extensive interactions with RNA and protein partners. Nevertheless, increasing understanding of post-transcriptional regulatory networks continues to expand therapeutic opportunities in this field.

**Table 1:** Therapeutic targeting of the TTP–HuR axis in cancer.

Strategy	Rationale	Practical example/outcome
Restore TTP	Reinforces decay of oncogenic and inflammatory ARE-containing transcripts.	In RAS-driven tumors, restoring TTP reduced PD-L1 mRNA and enhanced anti-tumor immunity
Inhibit p38MAPK/MK2	Prevents inhibitory phosphorylation of TTP.	Expected to reactivate endogenous TTP and blunt inflammatory signaling.
Reactivate ZFP36	Counteracts epigenetic or post-transcriptional repression of TTP.	Useful when TTP is silenced by promoter methylation or miRNA-mediated mechanisms.
Stabilize active TTP	Preserves the dephosphorylated, functional protein.	Aims to maintain TTP-mediated degradation of oncogenic transcripts.
Inhibit HuR	Blocks mRNA stabilization programs that support tumor growth and stress adaptation.	MS-444 inhibited colorectal cancer growth, increased apoptosis, disrupted HuR cytoplasmic trafficking, and reduced xenograft growth
		KH-3 suppressed breast cancer invasion and metastasis and reduced metastatic spread *in vivo*
Counter therapy resistance	Disrupts HuR-dependent DNA repair and survival networks.	HuR inhibition can sensitize aggressive tumors, including pancreatic cancer, to cytotoxic therapy
Dual targeting	Shifts the balance toward transcript decay.	Combining TTP restoration with HuR inhibition is particularly attractive in inflammation-driven tumors.

Therapeutic strategies targeting the TTP–HuR post-transcriptional axis in cancer.

Therapeutic strategies aim to restore TTP-mediated mRNA decay, inhibit HuR-driven transcript stabilization, or combine both approaches.

## Discussion

**Figure 2:**
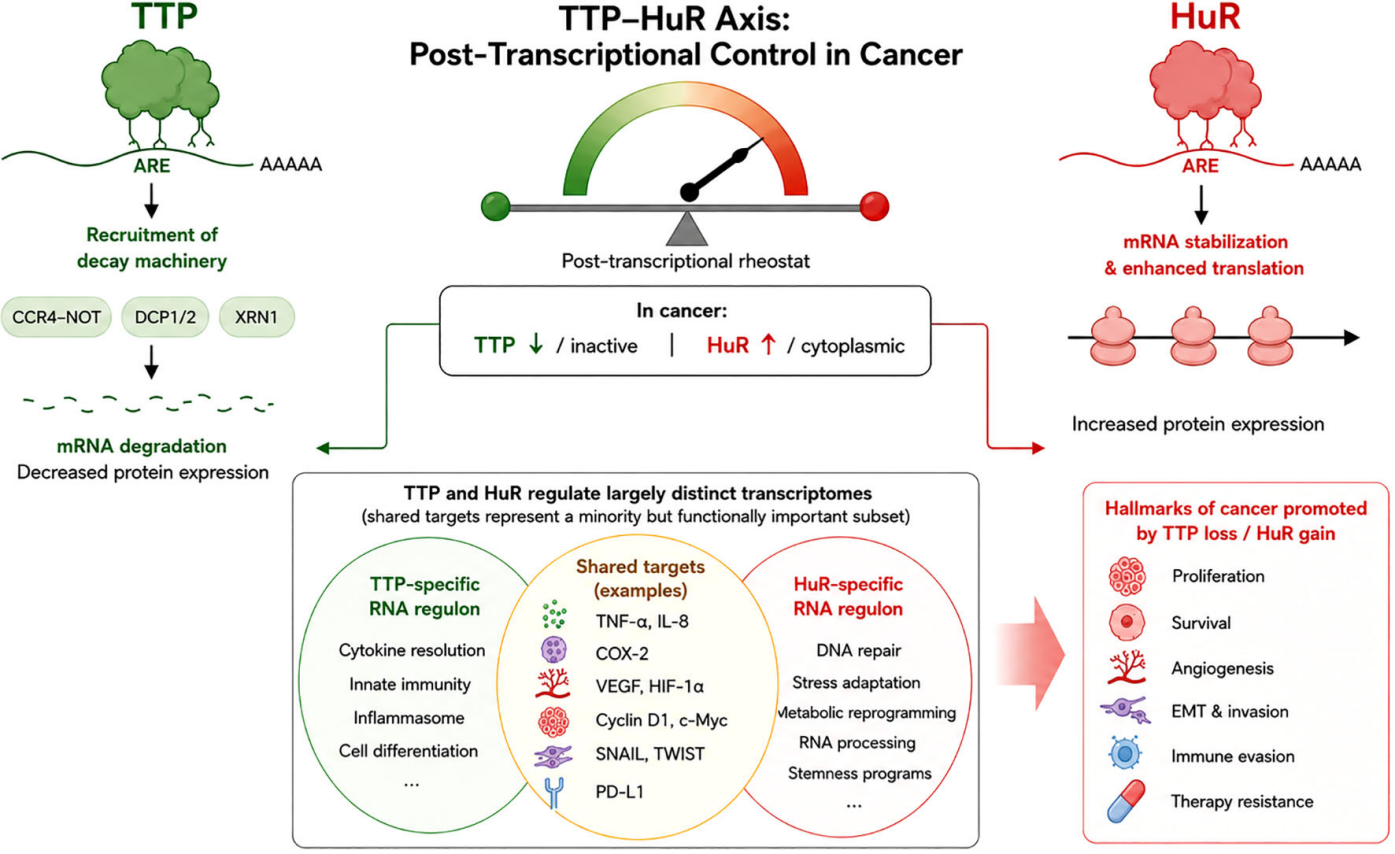
The TTP–HuR axis in cancer. Overview of the opposing functions of TTP and HuR in cancer. TTP promotes ARE-containing mRNA degradation through recruitment of decay machinery, whereas HuR stabilizes these transcripts and enhances translation. In cancer, reduced TTP activity and increased cytoplasmic HuR shift the balance toward oncogenic mRNA stabilization. The Venn diagram shows that TTP and HuR control largely distinct RNA regulons. The shared targets shown in the overlap represent selected examples of biologically important transcripts co-regulated by both proteins and constitute only a minority of their overall transcriptomes. Dysregulation of this axis contributes to multiple cancer hallmarks.

The study of TTP and HuR provides an interesting exemple of how post-transcriptional mechanisms contribute to cancer biology. Traditionally, these proteins have been viewed as functional antagonists operating on a common set of AU-rich element-containing transcripts. In this model, TTP promotes degradation of oncogenic and inflammatory mRNAs, whereas HuR stabilizes them, creating a post-transcriptional rheostat that influences proliferation, angiogenesis, invasion, and therapeutic resistance. Substantial experimental evidence supports this paradigm, particularly for transcripts such as TNF-α, IL-6, IL-8, COX-2, VEGF, HIF-1α, cyclin D1, c-Myc, SNAIL, TWIST and PD-L1. In many tumors, simultaneous reduction of TTP activity and increased cytoplasmic accumulation of HuR shift the balance toward stabilization of these transcripts, thereby promoting malignant progression. A graphical overview of the opposing functions of TTP and HuR in cancer is recapitulated in **Figure 2**. However, the biological relationship between TTP and HuR appears considerably more complex than a simple competition model. Although shared targets have received substantial attention because of their mechanistic relevance, current transcriptome-wide studies suggest that common targets represent only a fraction of the transcripts regulated by either protein. The biological significance of shared targets therefore derives less from their abundance than from their strategic position within regulatory networks controlling inflammation, angiogenesis, epithelial-to-mesenchymal transition, and stress adaptation. Indeed, HuR and TTP appear to regulate largely distinct RNA regulons that only partially overlap. HuR has been reported to associate with thousands of transcripts involved in DNA repair, cell-cycle progression, metabolic adaptation, hypoxic responses, RNA processing, and translational control. Many of these transcripts have never been identified as direct TTP targets. Conversely, TTP preferentially regulates transcripts involved in inflammatory resolution, cytokine production, innate immunity, and cellular differentiation, many of which are not known HuR substrates. Consequently, the effects of HuR activation cannot simply be interpreted as the mirror image of TTP loss, nor can TTP restoration be expected to reverse all HuR-mediated phenotypes. This distinction is particularly important when considering cancer progression. The classical model predicts that TTP loss and HuR activation cooperate because both events increase the abundance of shared oncogenic transcripts. While this mechanism contributes to malignant transformation, independent effects mediated through non-overlapping targets may be equally important. HuR-dependent regulation of DNA repair pathways, for example, may promote adaptation to genotoxic stress independently of TTP status. Likewise, TTP-mediated control of inflammasome components, cytokine resolution pathways, and innate immune signaling may influence tumor progression in ways that cannot be explained by HuR activity. Therefore, simultaneous TTP loss and HuR activation should be viewed not merely as two opposing events affecting the same transcripts, but as a broader reprogramming of post-transcriptional gene expression involving multiple interconnected yet partially independent RNA networks.

Another unresolved question concerns the extent to which TTP and HuR directly compete for binding to common transcripts *in vivo*. In fact, it remains unclear whether direct competition represents the dominant mechanism responsible for their antagonistic biological effects. Many observed phenotypes may instead arise indirectly through regulation of distinct upstream signaling pathways. For example, TTP-mediated destabilization of inflammatory mediators might alter signaling cascades that subsequently influence HuR localization and activity. Similarly, HuR-dependent stabilization of kinase, phosphatase, or transcription factor transcripts might affect TTP expression and phosphorylation. Thus, reciprocal regulation may occur at multiple levels extending beyond direct competition for AU-rich elements.

An additional layer of complexity derives from the fact that neither protein functions in isolation. Both TTP and HuR operate within dynamic ribonucleoprotein complexes that include additional RNA-binding proteins, microRNAs, long non-coding RNAs, deadenylases, translational regulators, and components of the RNA decay machinery. Moreover, emerging evidence indicates that epitranscriptomic regulation further contributes to this regulatory landscape by modulating RNA structure and the accessibility of RNA-binding proteins to their target transcripts, thereby integrating the TTP–HuR axis into a broader post-transcriptional regulatory network (**Figure 1**). The fate of any given transcript therefore depends not only on TTP or HuR abundance but also on the broader molecular environment in which these proteins act. Cell type, oncogenic mutations, inflammatory status, hypoxia, and metabolic conditions may all influence the composition of these complexes and ultimately determine transcript fate. The increasing availability of CLIP-seq, eCLIP, single-cell RNA sequencing, spatial transcriptomics, and quantitative proteomics is beginning to reveal the complexity of these regulatory networks.

From a therapeutic perspective, the increasing appreciation of the complexity of the TTP–HuR axis suggests that future strategies might move beyond the simple concept of globally activating TTP or inhibiting HuR. Although such approaches have shown encouraging experimental results, their efficacy is likely to depend on the molecular context of individual tumors, including the spectrum of expressed RNA-binding proteins, the abundance of shared and protein-specific target transcripts, and the activity of signaling pathways controlling TTP phosphorylation and HuR localization. A more comprehensive understanding of tumor-specific post-transcriptional regulatory networks will therefore be essential for the rational development of therapies targeting this axis.

One of the major challenges for future research remains the identification of the complete transcriptomes regulated by TTP and HuR in different cancer types and disease stages. While numerous shared targets have been characterized, current evidence indicates that these represent only a fraction of the transcripts regulated by either protein. Defining the relative contribution of common and protein-specific RNA networks will be important for understanding why alterations in TTP or HuR produce distinct biological effects across different tumor contexts. Integrating transcriptomic analyses with functional studies will help distinguish direct regulatory events from secondary consequences of altered signaling pathways. Technological advances are expected to substantially improve our understanding of these regulatory mechanisms. High-resolution RNA–protein interaction approaches, together with single-cell transcriptomics and spatial transcriptomic technologies, will make it possible to investigate TTP- and HuR-dependent regulatory programs at deeper resolution within individual cellular populations and distinct regions of the tumor microenvironment. These approaches should clarify how cancer cells, stromal cells and immune cells differentially exploit post-transcriptional regulation during tumor progression and therapeutic response. In conclusion, current evidence supports a model in which TTP and HuR function not simply as antagonistic regulators of a common pool of AU-rich element-containing transcripts, but as determinants of broader post-transcriptional regulatory networks. Their coordinated and context-dependent activities influence multiple hallmarks of cancer through both shared and distinct RNA targets. Continued investigation of these networks will undoubtedly provide new insights into cancer progression, treatment resistance and a possible new RNA-directed anticancer therapies.
